# Suppression of MyD88-dependent signaling alleviates neuropathic pain induced by peripheral nerve injury in the rat

**DOI:** 10.1186/s12974-017-0822-9

**Published:** 2017-03-31

**Authors:** Fan Liu, Zhiyao Wang, Yue Qiu, Min Wei, Chunyan Li, Yikuan Xie, Le Shen, Yuguang Huang, Chao Ma

**Affiliations:** 1 Department of Human Anatomy, Histology and Embryology, Institute of Basic Medical Sciences, Chinese Academy of Medical Sciences, School of Basic Medicine, Peking Union Medical College, Beijing, 100005 China; 2 Department of Anesthesiology, Peking Union Medical College Hospital, Chinese Academy of Medical Sciences, Peking Union Medical College, Beijing, 100730 China

**Keywords:** MyD88, TRIF, Dorsal root ganglion, Spinal dorsal horn, CCI, Neuropathic pain

## Abstract

**Background:**

MyD88 is the adaptor protein of MyD88-dependent signaling pathway of TLRs and IL-1 receptor and regulates innate immune response. However, it was not clear whether and how MyD88 and related signaling pathways in the dorsal root ganglion (DRG) and spinal dorsal horn (SDH) are involved in neuropathic pain.

**Methods:**

Chronic constriction injury (CCI) was used to induce neuropathic pain in the rat. The expression of MyD88, TRIF, IBA1, and GFAP was detected with immunofluorescent staining and Western blot. The expression of interleukin-1 beta (IL-1β), high mobility group box 1 (HMGB1), NF-κB-p65, phosphorylated NF-κB-p65, ERK, phosphorylated ERK, and tumor necrosis factor-alpha (TNF-α) was detected with Western blot. Pain-related behavioral effects of MyD88 homodimerization inhibitory peptide (MIP) were accessed up to 3 weeks after intrathecal administration.

**Results:**

Peripheral nerve injury significantly increased the protein level of MyD88 in the DRG and SDH, but had no effect on TRIF. MyD88 was found partly distributed in the nociceptive neurons in the DRGs and the astrocytes and microglia in the SDH. HMGB1 and IL-1β were also found upregulated in nociceptive pathways of CCI rats. Intrathecal application of MIP significantly alleviated mechanical and thermal hyperalgesia in the CCI rats and also reversed CCI-induced upregulation of MyD88 in both DRG and SDH. Further investigation revealed that suppression of MyD88 protein reduced the release of TNF-α and glial activation in the SDH in the CCI rats.

**Conclusions:**

MyD88-dependent TIR pathway in the DRG and SDH may play a role in CCI-induced neuropathic pain. MyD88 might serve as a potential therapeutic target for neuropathic pain.

**Electronic supplementary material:**

The online version of this article (doi:10.1186/s12974-017-0822-9) contains supplementary material, which is available to authorized users.

## Background

Neuropathic pain is associated with sensory abnormalities and altered stimulus-response function, such as allodynia, hyperalgesia, and loss of sensation in some areas [1, 2]. The International Association for the Study of Pain defined neuropathic pain as “pain caused by a lesion or disease of the somatosensory nervous system” (www.iasp-pain.org/Taxonomy#Neuropathicpain). Neuropathic pain poses a heavy burden on the quality of life of patients while currently available treatments are often ineffective. The underlying molecular and cellular mechanisms of neuropathic pain remain poorly elucidated. There are increasing evidences indicating a role of neuroimmune processes in the development of neuropathic pain [3–6].

The myeloid differentiation factor-88 adaptor protein (MyD88) is involved in Toll-like receptors (TLRs, except for TLR3) signaling and interleukin-1 receptor (IL-1R) signaling [7–10]. MyD88 mediates activation of TLRs or IL-1R and leads to NF-κB activation, inflammatory cytokines such as tumor necrosis factor-alpha (TNF-α) in immune cells [8–10]. TLRs are danger-associated and pathogen-associated molecular pattern receptors that regulate innate immunity via activated NF-κB-dependent and mitogen-activated protein kinase (MAPK)-dependent cytokine production [11, 12]. TLRs and IL-1R are not only expressed on immune cells but also found on sensory neurons in dorsal root ganglions (DRGs) and glial cells (microglia and astrocytes) in the spinal cord [5, 13–17]. A number of previous studies have found that TLRs in the spinal cord played an important role in the model of neuropathic pain and nerve injury, in which microglia and astrocytes produced proinflammatory cytokines by activating TLRs [18–20]. Nociceptors of DRG that express TLRs or IL-1R are also activated by LPS or inflammatory cytokine interleukin-1β, inducing pain hypersensitivity [5, 18–22]. Recent publications show that MyD88, adaptor protein of TLRs and IL-1R, is also found in the expression in DRG and spinal cord [18–20].We hypothesized that suppressed MyD88 adaptor protein in the DRG and spinal cord could alleviate peripheral nerve injury-induced neuropathic pain. Our findings reveal MyD88 adaptor protein involved in the neuropathic pain and may provide potential therapeutic strategies for treatment of neuropathic pain.

## Methods

### Animals

The adult male Sprague-Dawley rats (120–180 g) were used in our study. All animals were housed (4–5 rats per cage) in a standard 12-h light/dark cycle. These experiments were approved by the Institutional Animal Care and Use Committee in Chinese Academy of Medical Sciences, Institute of Basic Medical Sciences. Animals were randomly assigned to treatment or control groups.

### Animal model of neuropathic pain

In accordance with the study of Bennett and Xie [23], we performed chronic constriction injury (CCI) in the rats anesthetized through intraperitoneal injection of sodium pentobarbital (40 mg/kg) under aseptic condition. After the sciatic nerve of the mid-thigh level on right side was exposed, four snug ligatures of chromic gut suture were loosely tied around the nerve with about 1 mm space between the knots. The sciatic nerves of sham animals were exposed without ligation.

### Intrathecal catheterization and drug delivery

A PE10 catheter (length, 18 cm) was implanted intrathecally according to the protocol as described previously [24, 25]. Briefly, rats were anesthetized with sodium pentobarbital (40 mg/kg) and a 2-cm longitudinal incision was made above vertebrae L5–6.The catheter with guide wire was inserted in the hole that was made by a 23-gauge needle between lumbar vertebrae L5 and 6. The catheter was pushed through the intervertebral space until a sudden movement of tail or the hindlimb was observed and then passed gently 2 cm upward to reach the lumbar enlargement. The tip of the catheter was fixed on the neck area of the rats. After implantation, catheter localization was verified by intrathecal injection of lidocaine and all rats were observed for a minimum of 5 days before being performed with CCI surgery as described above. Rats showing motor weakness or without catheter in spinal space were excluded from the research. An intrathecal injection of 500 μM of MyD88 homodimerization inhibitory peptide (MIP, Novus Biologicals, CO, USA) or control peptide (Novus Biologicals, CO, USA) resolved in PBS was administrated on preoperative and postoperative days. A previous report showed that application of similar dose of MIP did not induce any significant change in locomotor function in the rats [18].

### Behavioral assessment of pain

All the behavioral measurements were carried out by an experimenter blinded to schedule during 10 am–3 pm. Mechanical allodynia was assessed by the up-down method using calibrated Electronic von Frey (Electronic von Frey 2393:II TC, USA). Rats were acclimated in suspended cages with wire mesh floor. The probe was applied perpendicularly to the paw with no acceleration at a force and held for approximately 6–8 s. An acute withdrawal of the paw was considered a positive response. Thermal hypersensitivity was tested using plantar test as described previously [24, 25]. The three measurements of mechanic threshold or thermal latency per side were averaged.

### Western blot analysis

The L4–6 spinal dorsal horn and DRGs were harvested from rats that were deeply anesthetized with sodium pentobarbital (40 mg/kg) and snap-frozen in liquid nitrogen. Tissues were homogenized on ice in RIPA buffer (20 mM Tris-HCl, 150 mM NaCl, 1% NP40, 1 mM EDTA, 0.5% Na deoxycholate, and 0.1% sodium dodecyl sulfate (SDS)) mixed with proteases inhibitor cocktail and phosphatases inhibitor cocktail (Sigma-Aldrich, MO, USA). After centrifuging, supernatants were collected and denatured with SDS-PAGE loading buffer consisting of 0.25 M Tris-HCl, 52% glycerol, 6% SDS, 5% β-mercaptoethanol, and 0.1% bromophenol blue for 5 min at 95 °C. Equal concentrations of lysates (30 μg) were separated by SDS-polyacrylamide gel electrophoresis gels and transferred to a PVDF membrane (GE Healthcare life science). After blocking with 5% BSA in TBST (Tris-buffered saline with 0.1%Tween 20) for 1 h at room temperature, membranes were incubated in TBST overnight at 4 °C with primary antibodies. The membranes were washed with TBST and then incubated with secondary antibodies diluted with 5% skim milk in TBST for 1 h at room temperature (Table 1 lists the primary and secondary antibodies used for Western blot analysis). Bands were detected with enhanced chemiluminescence reagents eECL Kit (CWBio, Beijing, China) using an ImageQuant LAS 4000 mini Chemiluminescence imaging analysis system (GE Healthcare life science, PA, USA), and the band densities were analyzed using ImageJ software (NIH, USA). For the detecting of total extracellular signal-regulated protein kinase (ERK) or total NF-κB protein, we again blocked the same member that was washed by Western blot antibody Stripping Buffer (CWbio, Beijing, China) with 5% BSA in TBST for 1 h at room temperature after detecting the phosphorylated ERK or phosphorylated NF-κB protein, incubated the member with primary antibodies (anti-total ERK or total NF-κB protein) overnight at 4 °C, and then incubated with secondary antibodies for 1 h at room temperature. Members that were re-incubated by primary and secondary antibodies were detected with enhanced chemiluminescence reagents eECL Kit using ImageQuant LAS4000 mini Chemiluminescence imaging analysis system.

**Table 1:** List of primary and secondary antibodies used for Western blot analysis

Antibody	Host	Company	Catalog number	Dilution	incubation conditions
MyD88 ^a^	rabbit	Abcam	ab2064	1:500	Overnight 4 °C
TRIF ^b^	rabbit	Abcam	ab13810	1:1000	Overnight 4 °C
IL-1β	rabbit	Proteintech	16806-1-AP	1:500	Overnight 4 °C
IBA1	rabbit	Proteintech	10904-1-AP	1:500	Overnight 4 °C
TNF-α	rabbit	Sino Biological	80045-RP02	1:500	Overnight 4 °C
GFAP	rabbit	Cell signaling technology	#12389	1:1000	Overnight 4 °C
HMGB1	rabbit	Cell signaling technology	#6893	1:1500	Overnight 4 °C
ERK	rabbit	Cell signaling technology	#4695	1:2000	Overnight 4 °C
pERK	rabbit	Cell signaling technology	#4370	1:1000	Overnight 4 °C
NF-κB	rabbit	Cell signaling technology	#8242	1:1000	Overnight 4 °C
pNF-κB	mouse	Cell signaling technology	#13346	1:1000	Overnight 4 °C
β-actin	mouse	Santa Cruz Biotechnology	TA-09	1:1000	Overnight 4 °C
Anti-rabbit IgG horseradish peroxidase (HRP)	Goat	ZSGB-BIO	ZDR-5306	1:3000	1 h RT
Anti-mouse IgG horseradish peroxidase (HRP)	Goat	ZSGB-BIO	ZDR-5307	1:3000	1 h RT

^a^ MyD88 antibody (Abcam, catalog #ab2064) was used for IHC or WB in 18 research articles (PubMed ID: 27389279, 26191134, 25446227, 26224622, 24307174, 24410883, 24477912, 24719558, 25187650, 23479602, 21278343, 20212458, 20837465, 20456021, 19553541, 17503341, 17114422, 16517734, 16461741)

^b^ TRIF antibody (Abcam, catalog #ab13810) was used for IHC or WB in 11 research articles (PubMedID: 27389279, 26191134, 24146036, 22361049, 20511556, 19265160, 19648648, 19389833, 17503341, 17314314, 17000866)

### Immunofluorescence staining

Rats were deeply anesthetized with sodium pentobarbital (40 mg/kg) and then perfused with warm saline followed by fresh 4% paraformaldehyde through the ascending aorta. The L4–6 spinal dorsal horn (SDH) and DRGs were collected, fixed in 4% paraformaldehyde for 4 h, and then dehydrated in 30% sucrose overnight at 4 °C. Tissues were mounted and finally cut to a thickness of 15 μm in a cryostat for immunofluorescent staining. After blocking in 10% normal donkey serum and 0.2% Triton X-100 in PBS for 1 h at room temperature, the tissue sections were incubated overnight at 4 °C in 10% normal donkey serum in PBS containing primary antibodies and then incubated with the proper secondary antibodies or Alexa Fluor 594-conjugated isolectin B4 (IB4) (1:100, Invitrogen/Thermo Fisher Scientific, USA) for 1 h. The slides were then washed in PBS and cover-slipped with VECTASHIELD Mounting Medium with DAPI. Table 2 lists the primary and secondary antibodies used for the immunofluorescence staining analyses. The percentages of positive neurons to total neurons were calculated and statistically analyzed. Images were captured using a confocal laser scanning microscope (FV1000, Olympus) and Olympus FluoView software. At least 12 fields of view (×200) from three sections on each ganglion were examined. DRG neurons were classified according to the cross-sectional areas of soma as small- (area <636 μm^2^), medium- (area 637–1431 μm^2^) and large-sized (area >1431 μm^2^). This classification is based on the criteria as previously described [26].

**Table 2:** List of primary and secondary antibodies used for Immunofluorescence staining

Antibody	Host	Company	Catalog ID	Dilution	incubation conditions
MyD88	rabbit	Abcam	ab2064	1:100	Overnight 4 °C
TRIF	rabbit	Abcam	ab13810	1:200	Overnight 4 °C
IBA1	goat	Abcam	ab107159	1:200	Overnight 4 °C
GFAP	goat	Abcam	ab53554	1:100	Overnight 4 °C
CGRP	goat	LifeSpan BioSciences	LS-C122785	1:500	Overnight 4 °C
anti-rabbit IgG Alexa Fluor 488	donkey	Jackson ImmunoResearch	711-545-152	1:400	1 h RT
anti-goat IgG Alexa Fluor 594	donkey	Jackson ImmunoResearch	705-585-147	1:400	1 h RT

### Statistical analysis

Values were expressed as group mean and standard errors (mean ± SEM) and analyzed using GraphPad Prism 6 and SPSS software (version 17.0). For analysis of Western blot, data were analyzed with one-way ANOVA followed by Student-Newman-Keuls test. Behavioral data were analyzed with two-way ANOVA. Chi-square tests were used to compare between two or more incidence of events. *P* < 0.05 was considered statistically significant.

## Results

### Up-regulation of MyD88 in nociceptive pathways after CCI

**Fig. 1:**
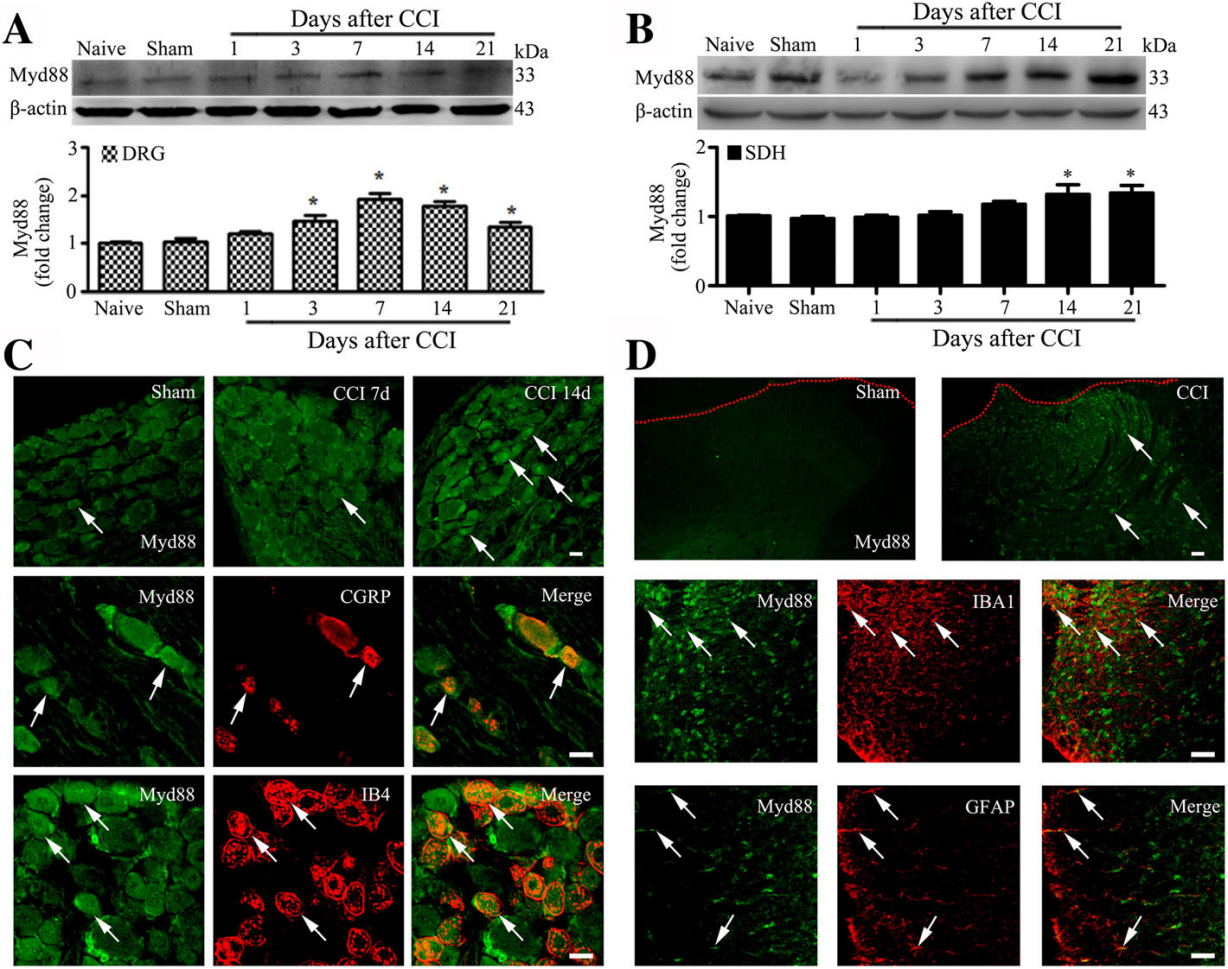
CCI increase expression MyD88 in rat DRG and SDH. **a** and **b** Western blot analyses the time course for MyD88 expression in DRG **(a)** and SDH **(b)** (*n* = 4 in each). Western blot analysis is shown on the top; quantification of protein levels (relative to naive group) is shown on the bottom. One-way ANOVA, *P < 0.05 versus naive and sham. **c** Cellular distribution of MyD88 in DRGs. MyD88 was expressed in the large-, medium-, and small-sized neurons (white arrows) sham to the CCI (top row). Double immunostaining shows coexpression of MyD88 with CGRP- and IB4-positive neurons, respectively (*middle and bottom rows*). **d** Distribution and cellular of MyD88 in the SDH. MyD88 was distributed predominantly in the superficial layers (*top row*). MyD88 was coexpression with astrocytes (GFAP, red) and microglial cells (IBA1, red) (*middle and bottom rows*). Scale bar: 20 μm (**c**, **d**)

First, we determined the time course of expression levels of MyD88 in DRGs and SDH after CCI in rats. Our Western blot results showed that CCI produced gradually rapid (in 3 days) and long-lasting (over 21 days) increase in the protein levels of in DRGs. MyD88 started increase from 3 days, peaked at 7 days, and maintained to the highest level at 21 days after CCI (Fig. 1a) in DRGs. And the protein levels of MyD88 were found produced and later increased to a high level from 14 to 21 days in the SDH after CCI operation (Fig. 1b).

Similar to the result of Western blot, we found that MyD88 were present at only very low levels in DRG and SDH of naïve and sham operation rats with immunofluorescence staining. Furthermore, by using immunofluorescence, the MyD88 protein immunoreactivity was found to be distributed in the three categories of neurons: large-sized, medium-sized, and small-sized neurons in DRG. We found a significant increase in the expression of MyD88 in DRG neurons (sham: 47.04%, 1049/2230; CCI 7 day: 67.30%, 1844/2740; CCI 14 day: 65.79%, 1579/2400) especially in small-sized neurons (sham: 55.34%, 974/1760; CCI 7 day: 77.86%, 1744/2240; CCI 14 day: 80.44%, 1448/1800) on days 7 and 14 after CCI in DRGs (Fig. 1c and Additional file 1: Figure S1A-B). The cellular localization of MyD88 in small neurons of DRGs was determined by double immunofluorescence. In the nociceptive small neurons, staining of MyD88 was colocalized in calcitonin gene-related peptide (CGRP)-positive and nonpeptidergic isolectin B4 (IB4)-positive nociceptive neurons of DRGs (Fig. 1c). As shown in the result of immunofluorescence, the expression of MyD88 was significantly upregulated in response to CCI operation in the SDH of rats. And the MyD88 protein was dominated and distributed in the superficial layers of the SDH. We also found that within 7 days after CCI, MyD88 immunoreactivity was colocalized with astrocytes (GFAP) and microglial cells (IBA1) (Fig. 1d). These results suggest that MyD88 signaling was activated in neurons of DRGs as well as SDH astrocytes and microglial cells after nerve injury.

### CCI increases expression of IL-1β and HMGB1 in nociceptive pathways

MyD88 is the downstream signaling adaptor protein of Toll/interleukin-1 receptor (TIR) signaling and is expressed in DRG and SDH. TLRs and IL-1R were also found to express on primary sensory neurons of DRG and glial cells in SDH [4, 5, 17, 21, 27, 28]. To define the mechanism of MyD88-dependent TIR signaling in CCI, we examined the expression of TLR2 and TLR4 endogenous ligand, high mobility group box 1 (HMGB1) and IL-1R endogenous ligand, interleukin-1 beta (IL-1β).

**Fig. 2:**
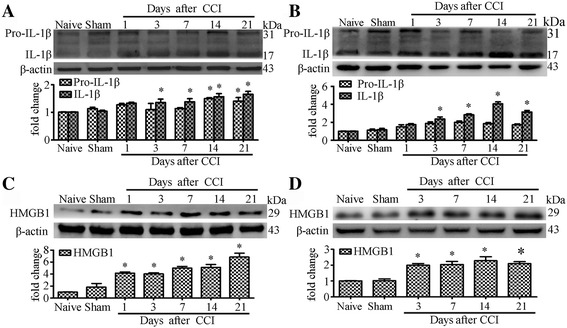
CCI increase expression IL-1β and HMGB1 in rat DRG and SDH. **a** and **b** Western blot analyses the time course for Pro-IL-1β and IL-1β expression in DRG (A) and SDH (B). **c**-**d** Western blot analyses the time course for HMGB1 expression in DRG (C) and SDH (D). Western blot analysis is shown on the top; quantification of protein levels (relative to naive group) is shown on the bottom. *N* = 4 in each group, One-way ANOVA, *P < 0.05 vs. naive or sham

Western blot showed CCI significantly increased protein levels of both pro-IL-1β and IL-1β in the DRG (Fig. 2a). In the SDH, CCI induced significant upregulation of the protein levels (from day 3 to day 7 after CCI) of IL-1β, not pro-IL-1β (Fig. 2b). We then examined the protein levels of HMGB. After CCI, protein expression levels of HMGB1 in the DRG increased rapidly and significantly from 1 day to lasting 21 days (Fig. 2c). The levels of HMGB1 also greatly increased in the SDH of CCI rat model (Fig. 2d).

### Regulation and activation of NF-κB signal and ERK signal in nociceptive pathways of CCI rats

**Fig. 3:**
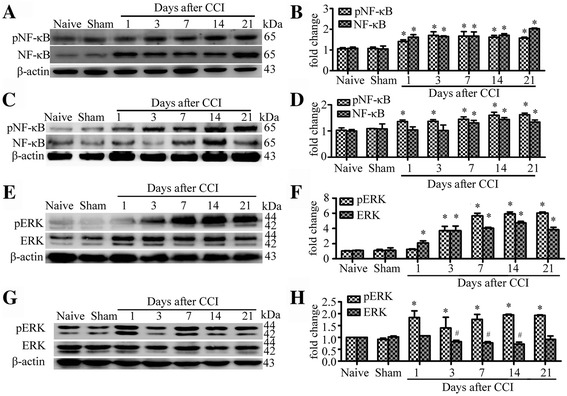
CCI induce NF-κB p65 and ERK signaling activation in rat DRG and SDH. **a**-**b** Western blot analysis shows time course for the expression of total NF-κB p65 and activated phospho-NF-κB p65 in DRG (A). Quantification of protein levels of total NF-κB p65 and activated phospho-NF-κB p65 in DRG (B). One-way ANOVA, *P < 0.05 versus naive and sham. **c-d** Western blot analysis shows time course for the expression of total NF-κB p65 and activated phospho-NF-κB p65 in SDH (C). Quantification of protein levels of total NF-κB p65 and activated phospho-NF-κB p65 in SDH (D). **e-f** Western blot analysis shows time course for the expression of total ERK and activated pERK in DRG (E). Quantification of protein levels of total ERK and activated pERK in DRG (F). **g-h** Western blot analysis shows time course for the expression of total ERK and activated pERK in SDH (G). Quantification of protein levels of total ERK and activated pERK in SDH (H). *N* = 4 in each group, One-way ANOVA, *P < 0.05 vs. naive or sham

The protein levels of both total NF-κB p65 and activated phospho-NF-κB p65, in L4 and L5 DRGs, increased significantly compared with naïve and sham groups. We detected rapid and long lasting increase of the expression of total and activated phospho-NF-κB p65 in DRG (Fig. 3a-b). Both total NF-κB p65 and activated phospho-NF-κB p65 levels were found to peak at 3 days after CCI and remained elevated to the 21 days, and did not recover to the control level of naïve and sham after CCI. Similar to the changes on DRGs, activated phospho-NF-κB p65 as well as total NF-κB p65 also significantly increased in SDH after CCI. Phospho-NF-κB p65 were found to increase significantly and persistent on all time points from 1 day to 21 days of CCI-induced nerve injure compare with control group. Our results demonstrated that total NF-κB p65 did not increase rapidly but showed an obvious increase at 7 to 21 days compared with naïve and sham groups in SDH after CCI (Fig. 3c-d). The expression of total NF-κB p65 on SDH maximized at day 14 and maintained elevated to day 21after CCI-induced nerve injury.

Extracellular signal-regulated protein kinase 1/2 (ERK) signal pathway was involved in TLR-mediated proinflammatory response. We found the protein levels of both ERK1 (p44 MAPK, 44 kDa) and ERK2 (p42 MAPK, 42 kDa) increased significantly on all time points from 1 to 21 days after CCI in DRG (Fig. 3e, f). Western blot analysis also revealed activation of ERK in DRG and SDH after CCI. A marked and persistent increase of both phospho-ERK1 and phospho-ERK2 were observed in DRG (3 to 21 days) and SDH (1 to 21 days) after CCI (Fig. 3e–h).

### MIP treatment relieve neuropathic pain after CCI

**Fig. 4:**
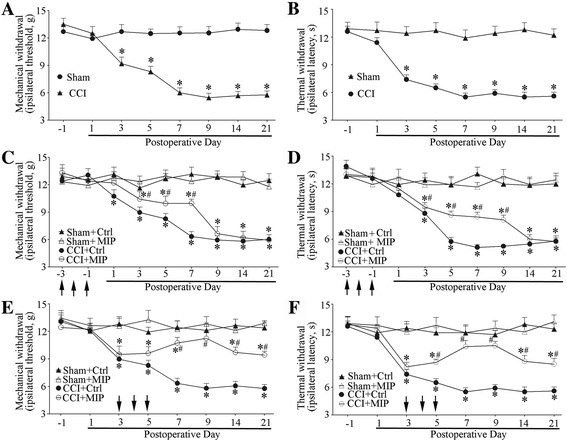
Attenuated neuropathic pain by of MIP after CCI treatment. **a-b** CCI-induced mechanical allodynia (**a**) and thermal hyperalgesia (**b**). Two-way ANOVA, *P < 0.05 versus sham. **c-d** Pre-intrathecal injection of MIP attenuated CCI-induced mechanical allodynia (**c**) and thermal hyperalgesia (**d**) (each administration is indicated by an arrow on the 3, 2, and 1 days before CCI). **e-f** Mechanical allodynia (**e**) and thermal hyperalgesia (**f**) is attenuated by MIP in the early phase of CCI operation (each administration is indicated by an arrow on the 3, 4, and 5 days after CCI). MIP (500 mM) was administrated i.t.in a volume of 20 μl; The control peptide was used in the control (Ctrl) group. Nine rats were included in each group. Two-way ANOVA, *P < 0.05 vs. Sham + Ctrl or Sham + MIP, #*P* < 0.05 vs. Sham + Ctrl, Sham + MIP or CCI + Ctrl

Compared with sham group, rats with CCI showed a decrease in paw mechanical withdrawal threshold at postoperative day (POD) + 3, achieving a significant difference at POD7, getting maximal at POD14 and thus maintained throughout POD21 (Fig. 4a). The paw thermal withdrawal latency of CCI group rats also decreased significantly at POD7 and POD14 compared to that of sham group (Fig. 4b). To determine the role of MyD88-dependent signaling of TIR in CCI, we used MIP to inhibit MyD88 dependent signaling. MIP was delivered into the cerebrospinal fluid of lumbar enlargement by intrathecal injection (500 μM in a volume of 20 μl in rats).

We intrathecally administrated MIP and control peptide daily from preoperative day −3 to day −1 (before CCI). The results showed that MIP administration on preoperative period delayed CCI-induced mechanical allodynia and thermal hyperalgesia, with no effects in sham operative rats (Fig. 4c, d). Then we injected MIP and control peptide intrathecally daily for three consecutive days (POD 3–5). We found that MIP administration reversed the CCI-induced neuropathic pain and produced long-lasting alleviative effects (POD 7–21) on the mechanical allodynia and thermal hyperalgesia (Fig. 4e, f).

### MIP treatment decrease expression level of MyD88 protein after CCI

**Fig. 5:**
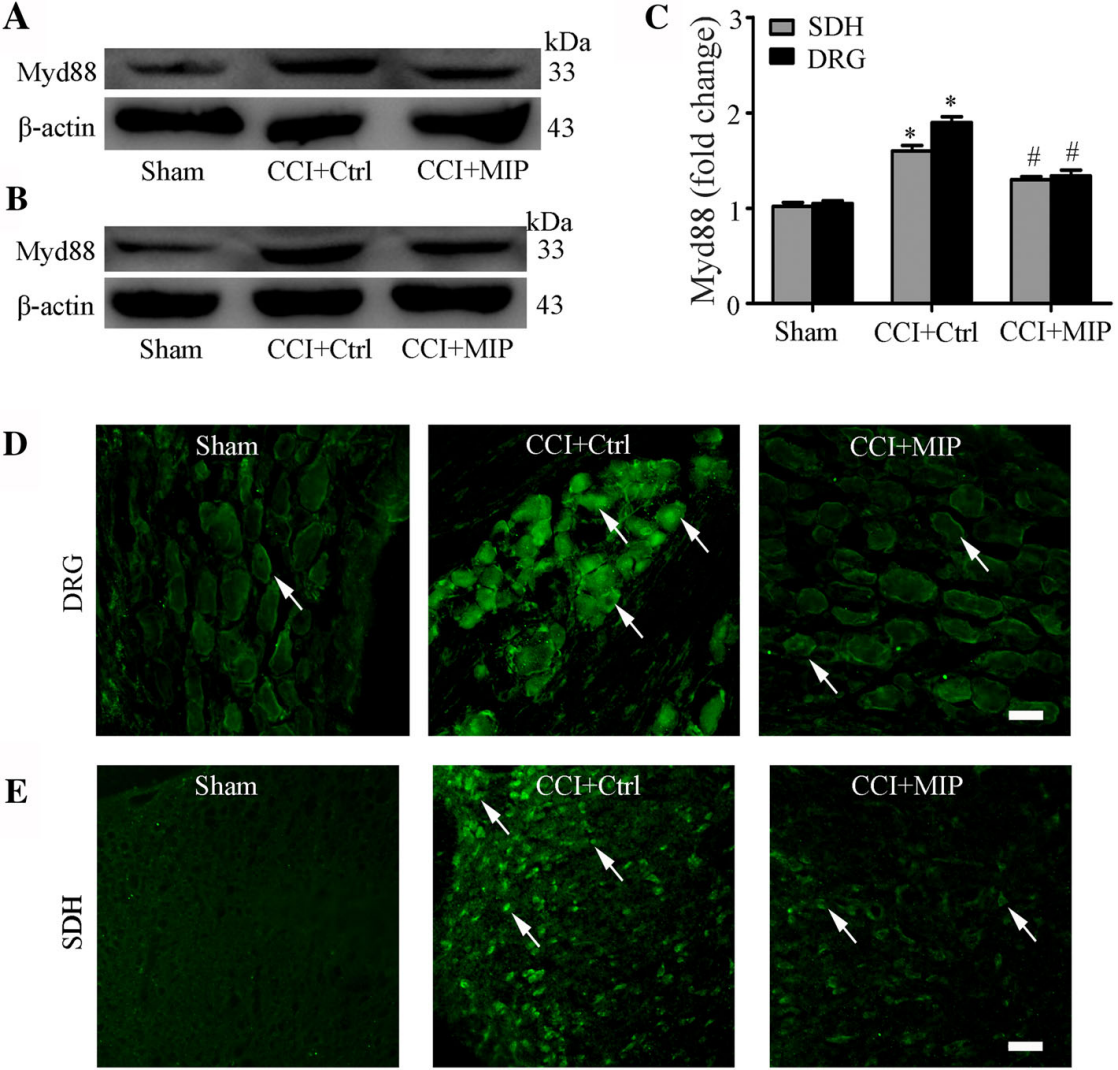
Intrathecal administration of MIP decreases MyD88 protein expression in rat DRG and SDH after CCI. **a-b** Western blot showing inhibitory effects of MIP on CCI-increased protein level of MyD88 in DRG (A) and SDH (B). **c** Data summary of B. **d-e** Immunofluorescence showing inhibitory effects of MIP on expression of MyD88 in DRG (D) and SDH (E). Scale bar: 20 μm (**d**, **e**). MIP (i.t., in a volume of 20 μl, 500 mM) was given once a day on postoperative days 3, 4, and 5, respectively. The control peptide was used in the control (Ctrl) group. Tissues were collected on postoperative days 14 (n = 4 each group).One-way ANOVA, *P < 0.05 vs. Sham, #*P* < 0.05 vs. CCI + Ctrl

Given that MyD88 expression increased significantly and MyD88-NF-κB p65 signaling pathways activated postoperatively, we examined whether MyD88-dependent signaling pathways could be suppressed by MIP treatment, which relieved neuropathic pain in the CCI rats. Our Western blot and immunofluorescent results showed that the expression of MyD88 was significantly decreased in the L4–5 DRGs and SDH at day 14 after intrathecally administered MIP to the CCI rats in comparison to rats that received the control peptide (Fig. 5a–e and Additional file 1: Figure S1C).

### Inhibition MyD88 suppresses activation of NF-κB signal and ERK signal after CCI

**Fig. 6:**
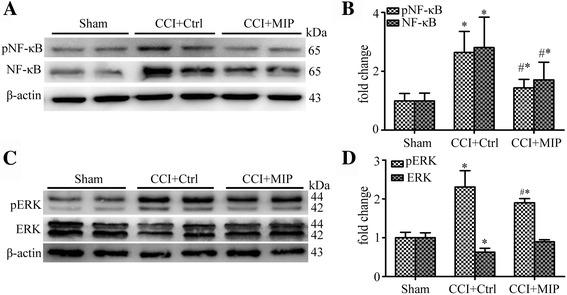
Suppressed of NF-κB p65, ERK signal by inhibition of Myd88 in rat SDH after CCI. **a** Western blot showing inhibitory effects of MIP on CCI-induced increased protein level of NF-κB p65 and pNF-κB p65. **b** Data summary of A. **c** Western blot showing inhibitory effects of MIP on CCI-induced increased protein level of ERK and pERK. **d** Data summary of C. Others are the same as Fig. 5

NF-κB p65 and ERK signaling pathways were activated at postoperation, we examined whether suppressed MyD88 by MIP treatment could alter the activated situation of NF-κB p65 and ERK proteins in rats of CCI-induced nerve injury. Our Western blot results showed that the MIP treatment significantly decreased the phosphorylation of NF-κB p65 and ERK proteins in CCI-rat SDH at day 14 after intrathecally administered MIP on CCI rats in comparison to rats that received the control peptide (Fig. 6a–d).

### Inhibition MyD88-dependent pathway suppresses the production of TNF-α and activation of astrocyte and microglial cell

**Fig. 7:**
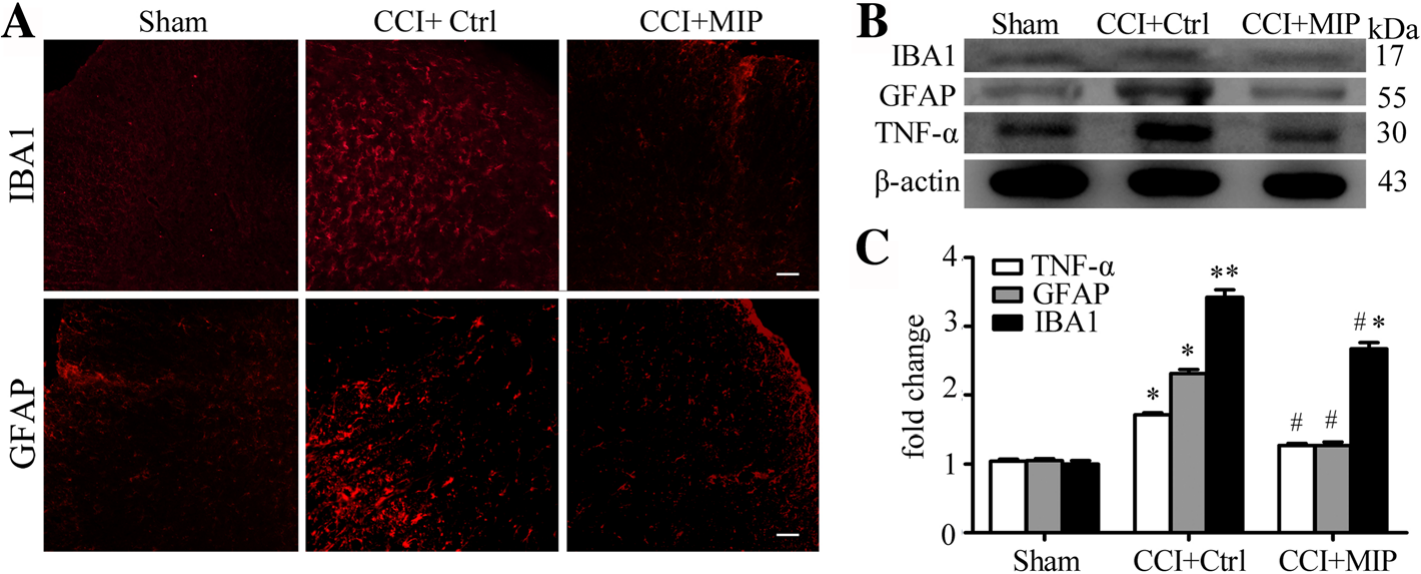
Suppressed activation of glial cells and TNF-α production by inhibition of MyD88 in rat SDH after CCI. **a** Immunostaining showing inhibitory effects of MIP on activation of microglial cells (IBA1), and astrocytes (GFAP). Scale bar: 20 μm. **b** Western blot showing inhibitory effects of MIP on CCI-induced increased protein level of IBA1, GFAP, and TNF-α. **c** Data summary of B. Others are the same as Fig. 5

Glial cells, including astrocytes and microglial cells, play a critical role in the initiation and maintenance of neuropathic pain [4, 15, 29]. Nerve injury-induced activation of astrocytes and microglial cells act as modulators in the maintenance of central sensitization. GFAP is an astrocyte-specific marker that distinguishes from other glial cells and also activation marker of astrocytes in the CNS. IBA1 play as specific activated marker of microglia in the CNS. Immunofluorescent results showed CCI-induced activation of astrocytes (GFAP) and microglia (IBA1) were suppressed by intrathecal injection of MIP (500 μM on POD 3–5) (Fig. 7a). CCI-induced increased expression of GFAP and IBA1 protein in the spinal cord SDH were also reduced by the administration of MIP (Fig. 7b, c).

We also examined the expression of proinflammatory cytokines TNF-α of SDH. After nerve injury, the TNF-α was upregulated and played an important role in the maintenance of neuropathic pain [30]. The significantly increased protein levels of TNF-α induced CCI were suppressed greatly by intrathecal injection of MIP in the SDH (Fig. 7b, c). These results suggested that MyD88-dependent TIR signaling played an essential role in the development of neuropathic pain.

### Unchanged expression and distributions of TRIF in DRG and SDH after CCI

Toll-like receptor signaling pathways were activated via MyD88-dependent signaling transduction and MyD88-independent signaling transduction pathway [8, 13]. Toll-receptor-associated activator of interferon (TRIF) was the adaptor protein of MyD88-independent signaling transduction pathway [7]. The results of Western blot were observed that CCI had no effect on the protein level of TRIF compared with naïve and sham operation in L4–5 DRGs and SDH of rats (Additional file 1: Figure S2A-B and Additional file 1: Figure S4A-B). The protein presented in the neurons of DRGs and co-expressed partly with CGRP and IB4-positive neurons (Additional file 1: Figure S2C). In SDH, we found that TRIF distributed in the superficial layers. And astrocytes and microglial cells partly expressed TRIF protein on SDH (Additional file 1: Figure S2D). After CCI, unchanged TRIF protein of DRGs and SDH may indicate that MYD88-independent signaling transduction of DRGs and SDH was not activated.

In addition, we performed experiments to test whether MIP treatment could also decrease the expression of TRIF in DRG and SDH. However, our Western blot and immunofluorescent data showed that the expression of TRIF was not affected in the DRG and SDH at POD 14 in MIP treatment CCI rats (Additional file 1: Figure S3A-E and Figure S4C).

## Discussion

In the present study, we explored the role of MyD88-dependent TIR signaling pathways in the DRG and SDH in a rat model of CCI-induced neuropathic pain. We found that CCI induced a rapid and long-term upregulation of MyD88 in the DRG and SDH of rat. Meanwhile, the proinflammatory cytokine IL-1β and HMGB1 were upregulated in the DRG and SDH. We also found that phospho-NF-κB p65 and phospho-p44/42 MAPK of Toll/interleukin-1 receptor downstream signal protein were rapidly and long-lastingly upregulated in the DRG and SDH after CCI. In contrast, CCI did not cause any significant change in TRIF. Intrathecal injection of MyD88 inhibitor MIP attenuated CCI-induced pain and decreased MyD88 expression in DRG and SDH. Inhibition of MyD88 suppressed the phospho-NF-κB p65, phospho-p44/42 MAPK, the production of TNF-α, and the activation of astrocytes and microglial cells in SDH. These results suggested that MyD88-dependent TIR/NF-κB p65 and p44/42 MAPK pathway, activated by IL-1β and HMGB1, was involved in CCI-induced neuroinflammation and neuropathic pain. In this study, we administrated MIP on preoperative days −3 to −1 (before CCI) to achieve a maximum and sustaining effect of the drug. Previous studies used a similar strategy of MIP application to inhibit MyD88 in vitro or in vivo [31–33].

HMGB1 is a proinflammation mediator and endogenous ligand of TLRs such as TLR2 and TLR4 [34]. HMGB1 can be induced in the DRG and/or SDH in multiple animal models of pain [34–36]. IL-1β is a potent proinflammatory cytokine and endogenous ligand of IL-1R [21]. HMGB1 and IL-1β can activate calcium mobilization in DRG neurons and stimulate astrocytes or microglia cells to produce proinflammatory mediators or cytokines such as TNF-α via activation of NF-kappa-B, MAPK, or other pathways [35, 37]. Previous studies showed that intrathecal injection of HMGB1 or IL-1β induced pain hypersensitivity including heat hyperalgesia and mechanical allodynia [34, 37]. Release of HMGB1 and IL-1β in the nociceptive pathway may play a crucial role for the development of pain via influencing adjacent neurons and glia [38]. In this study, we also found that HMGB1 and IL-1β was robustly produced and sustained through POD 21 in the DRG and SDH of CCI rats.

TLRs play a pivotal role in innate immune responses. Increasing evidence suggests that TLRs are expressed in primary sensory neurons in DRG and TG [5]. TLR4 mainly express in small-diameter sensory neurons of DRG and TG, which are mostly nociceptive sensor and regulate nociceptive sensation such as pain [16, 20, 21]. Functional TLRs including TLR2 and TLR4 also express in microglia and astrocytes that modulate glial activation and spinal inflammatory of the spinal injury or chronic pain-induced central sensitization [17, 39]. TLR4 mediates the hyperalgesia and neuroinflammation by damage/pathogen-associated molecular pattern components such as heat shock protein 90 (HSP90), HMGB1, and LPS [16, 21, 34, 35, 40]. Peripheral neurons of DRG or glial cells of spinal cord may directly produce HMGB1 and/or HSP90 when peripheral and/or central nervous system are damaged or administrated with drugs [18, 34, 35]. In this study, we measured total HMGB1 by Western blots, while only disulfide HMGB1 is known to be a TLR4 agonist [41, 42]. This problem will be addressed in future studies. In addition, a recent study also found that HMGB1 activates pro-inflammatory signaling via TLR5, leading to allodynia [43]. Further experiments using specific TLR2 or TLR4 antagonists or knockout mice could be performed to determine their roles in the MyD88-dependent signaling pathway.

IL-1R was an important receptor for regulating immune responses and inflammation [44]. It expresses in nociceptive neurons of DRG and glial cells of SDH and mediates interleukin-1 including interleukin-1β-induced activation of cell [27, 28, 45]. IL-1β can act directly on primary sensory neurons to evoke excitatory action on nociceptor neurons by IL-1R [28]. IL-1β also activates IL-1R to contribute to hyperalgesia and the establishment of peripheral and central sensitization [28, 46].

Two distinct signaling transduction pathways of TLRs were found [13]. Activated TLRs and/or IL-1R signaling are involved in the recruitment of adaptor molecules such as MYD88 or TRIF, then phosphorylate NF-kappa-B or MAPK via IL-1 receptor-associated kinase1 (IRAK1) and TNF receptor-associated factors6 (TRAF6) [7, 9, 10, 13]. MyD88 is the adaptor protein of Toll/interleukin-1 receptor (TIR) and plays an important role in the trafficking of TIR signal pathway [7, 10]. MyD88 is expressed in DRG neurons and glial cells and could be significantly increased in the DRG from rat models of chronic pain induced by chemotherapy [18]. SNL lesions produce chronic pain is approximately 50% reduced in MyD88-deficient mice [19, 47]. Consistently, intrathecal administration of MyD88 inhibitor suppresses pain of paclitaxel-induced peripheral neuropathy when applied 14 days after paclitaxel administration [18]. TRIF is an adaptor protein of the MyD88-independent signaling transduction pathways of TLRs and shares mostly by TLR3 and TLR4 signaling [13]. The SNL-induced allodynia was significantly alleviated in the MyD88 knock-out mice with reduced glial activation in SDH and ATF3 expression in the DRG, but these effects were not observed in the TRIF deficient mice [47]. TRIF expression was found not significantly changed in the SDH in a rat model of paclitaxel-induced peripheral neuropathic pain [18]. Our results also showed a lack of change in TRIF protein in the DRG and SDH of CCI rats. Detailed mechanisms for the upregulation and activation of MyD88 without increases in TRIF with TLR signaling should be further investigated in the future.

## Conclusions

**Fig. 8:**
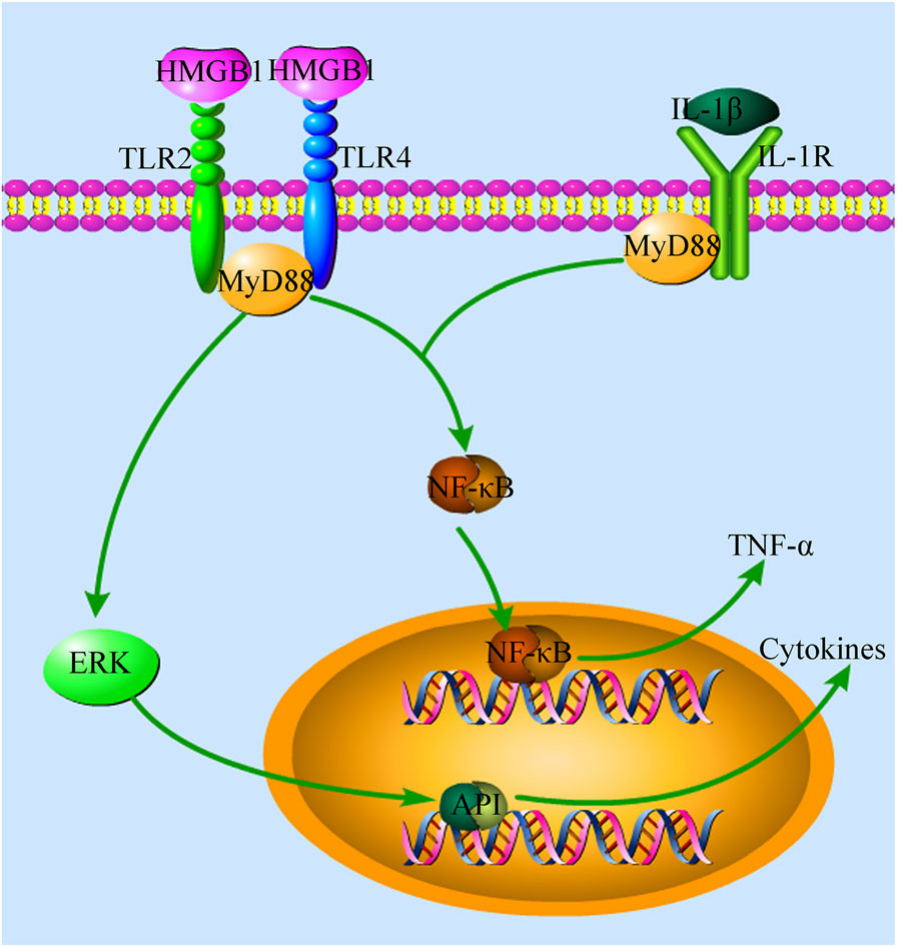
Schematic illustration demonstrates MyD88-dependent signaling pathways of neuropathic pain induced by CCI. Nerve injury produces abundant HMGB1and IL-1β in the DRG and SDH. The binding of HMGB1 and IL-1β to their receptors (TLR2/4 andIL-1R, respectively) activates MyD88 in the DRG and SDH, which phosphorylate NF-κB p65 and ERK. Phosphorylated NF-κB p65 subsequently enter the nucleus to regulate the expression of proinflammation cytokines such as TNF-α. Phosphorylated ERK enter the nucleus to induce transcription factors such as AP-1, which regulates the expression of certain cytokines and activates glial cells. All these signaling events consequently result in central and peripheral sensitizations that produce neuropathic pain

In this study, we found that peripheral nerve injury triggered the upregulation of IL-1β and HMGB1, which could bind to their receptors in the DRG and SDH and transmit signals via adaptor protein MyD88. MyD88 may subsequently act through phosphorylated NF-κB and ERK to regulate the expression of proinflammation cytokines and activate glia cells. MyD88-dependent TIR pathway may play a role in CCI-induced neuroinflammation and neuropathic pain. A schematic illustration for the proposed MyD88-dependent signaling pathways of neuropathic pain was provided in Fig. 8 This study might suggest a potential strategy for the treatment of neuropathic pain targeting MyD88.

## Additional file

Additional file 1: Figure S1. Quantification analysis from Immunofluorescence staining results of MyD88 in DRG neurons. **Figure S2** Unchanged expression and cellular distributions of TRIF protein in rat DRG and SDH after CCI. **Figure S3** Protein level of TRIF by intrathecal administration of MIP in rat DRG and SDH after CCI. **Figure S4** Quantification analysis from Immunofluorescence staining results of TRIF-positive neurons in DRGs.

## Abbreviations

CCI: Chronic constriction injury
CGRP: Calcitonin gene-related peptide
CNS: Central nervous system
DRG: Dorsal root ganglion
ERK: Extracellular signal-regulated protein kinase
GFAP: Glial fibrillary acidic protein
HMGB1: High mobility group box 1
IB4: Isolectin B4
IBA1: Allograft inflammatory factor 1
IL-1β: Interleukin-1 beta
IRAK1: IL-1 receptor-associated kinase1
MAPK: Mitogen-activated protein kinase
MIP: MyD88 homodimerization inhibitory peptide
MyD88: Myeloid differentiation primary response protein 88
NF-κB: Transcription factors of the nuclear factor κB
POD: Postoperative day
SDH: Spinal dorsal horn
TIR: Toll/interleukin-1 receptor
TLR: Toll-like receptor
TNF-α: Tumor necrosis factor-alpha
TRAF6: TNF receptor-associated factors 6
TRIF: TIR-domain-containing adaptor-inducing beta interferon
