# Supplementary material for: Suppression of MyD88-dependent signaling alleviates neuropathic pain induced by peripheral nerve injury in the rat
Source: J Neuroinflammation. 2017 Mar 31;14:70. doi: 10.1186/s12974-017-0822-9 (PMC5374701; doi:10.1186/s12974-017-0822-9)
Supplement: Additional file 1: Figure S1. — Quantification analysis from Immunofluorescence staining results of MyD88 in DRG neurons. Figure S2 Unchanged expression and cellular distributions of TRIF protein in rat DRG and SDH after CCI. Figure S3 Protein level of TRIF by intrathecal administration of MIP in rat DRG and SDH after CCI. Figure S4 Quantification analysis from Immunofluorescence staining results of TRIF-positive neurons in DRGs. [file 12974_2017_822_MOESM1_ESM.docx]

**Additional file 1**

**
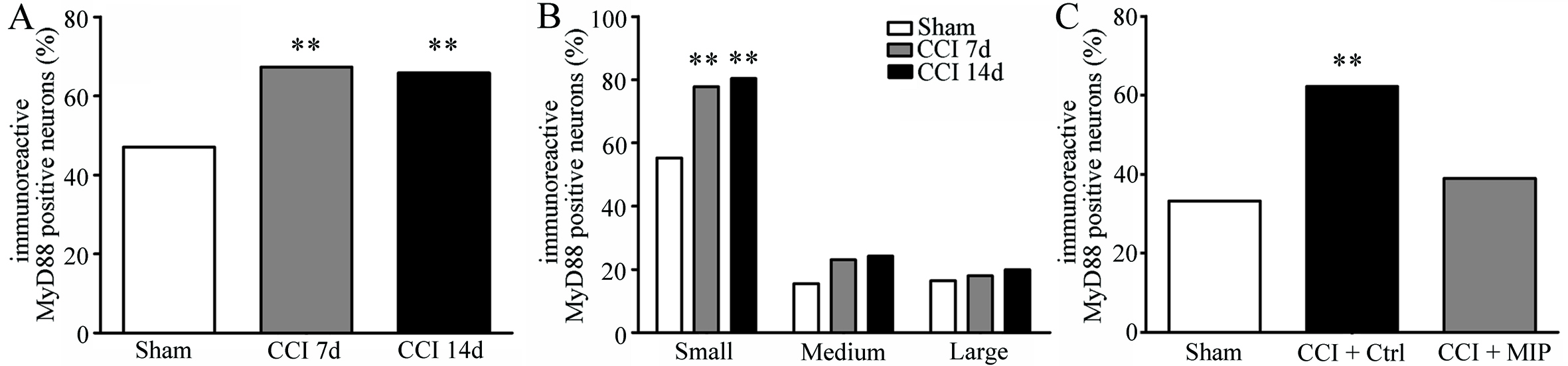
**

**Supplementary Figure S1** Quantification analysis from Immunofluorescence staining results of MyD88 in DRG neurons. (A) Percentage of MyD88-positive neurons in DRGs of sham, CCI (7d) and CCI (14d) rats. (B) Size distribution of MyD88-positive neurons in DRGs of sham, CCI (7d) and CCI (14d) rats. Chi-square test in A-B (n > 100 neurons from 4 rats in each group), ***P* < 0.01, CCI (7d) and CCI (14d) versus Sham. (C) Percentage of MyD88-positive neurons in small-sized DRG neurons from sham, CCI + Ctrl and CCI + MIP rats. Chi-square test in C (n > 100 neurons from 4 rats in each group), ***P* < 0.01, CCI +Ctrl vs. Sham or CCI + MIP.

**
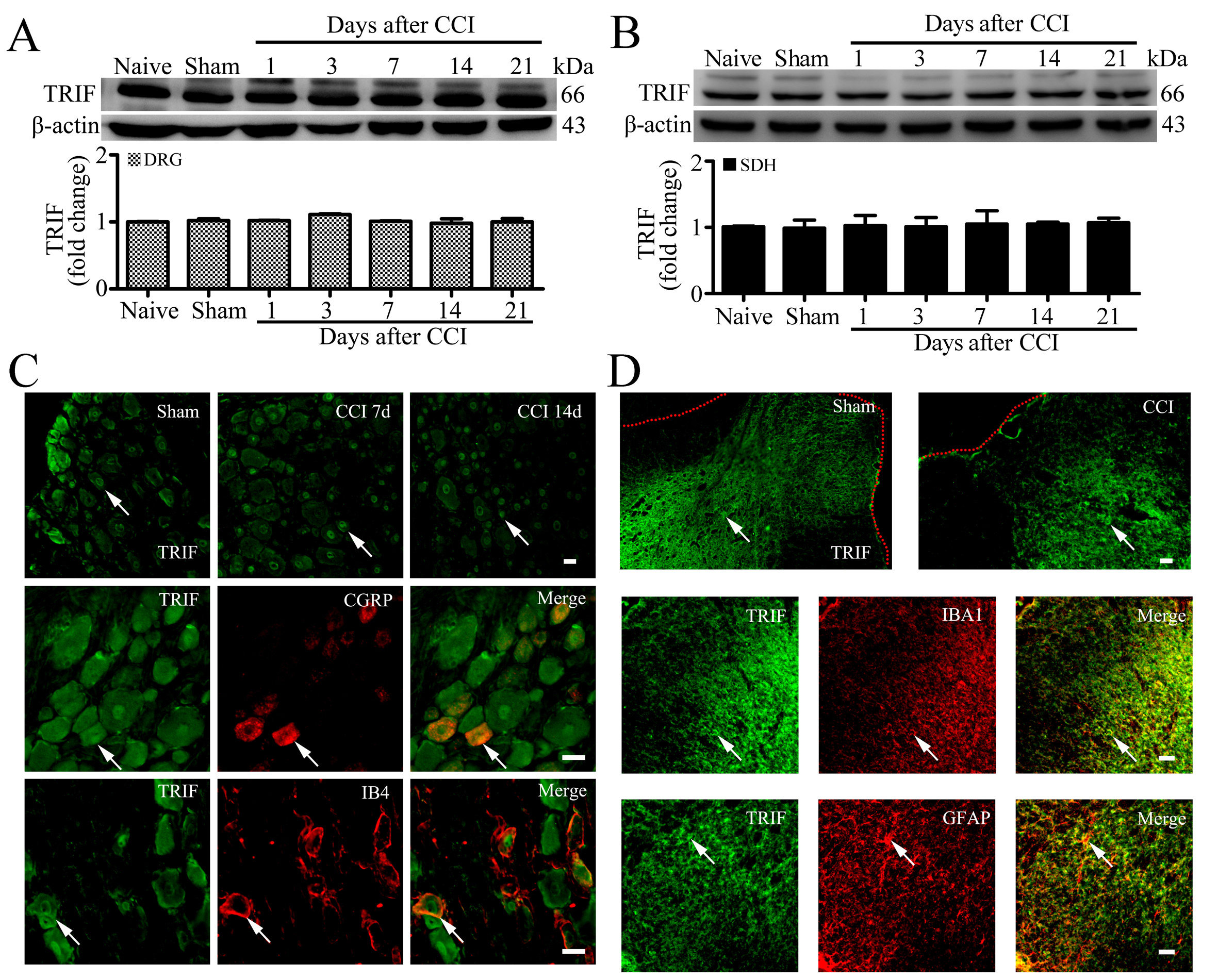
**

**Supplementary Figure S2** Unchanged expression and cellular distributions of TRIF protein in rat DRG and SDH after CCI. **(A and B)** Western blot analyses the time course for TRIF expression in DRG **(A)** and SDH **(B)** (*n* = 4 in each). Western blot analysis is shown on the top; quantification of protein levels (relative to naive group) is shown on the bottom, One-way ANOVA. **(C)** Cellular distribution of TRIF in DRGs. TRIF was expressed in the large-, medium-, and small-sized neurons (white arrows) sham to the CCI (top row). TRIF was coexpressed with CGRP- and IB4-positive neurons, respectively (middle and bottom rows). Scale bar: 20 μm. **(D)** Distribution and cellular of TRIF in the SDH. TRIF was distributed predominantly in the superficial layers (top row). TRIF was coexpressed with IBA1 (microglia, red, middle row) and GFAP (astrocytes, red, bottom rows). Scale bar: 20 μm.

**
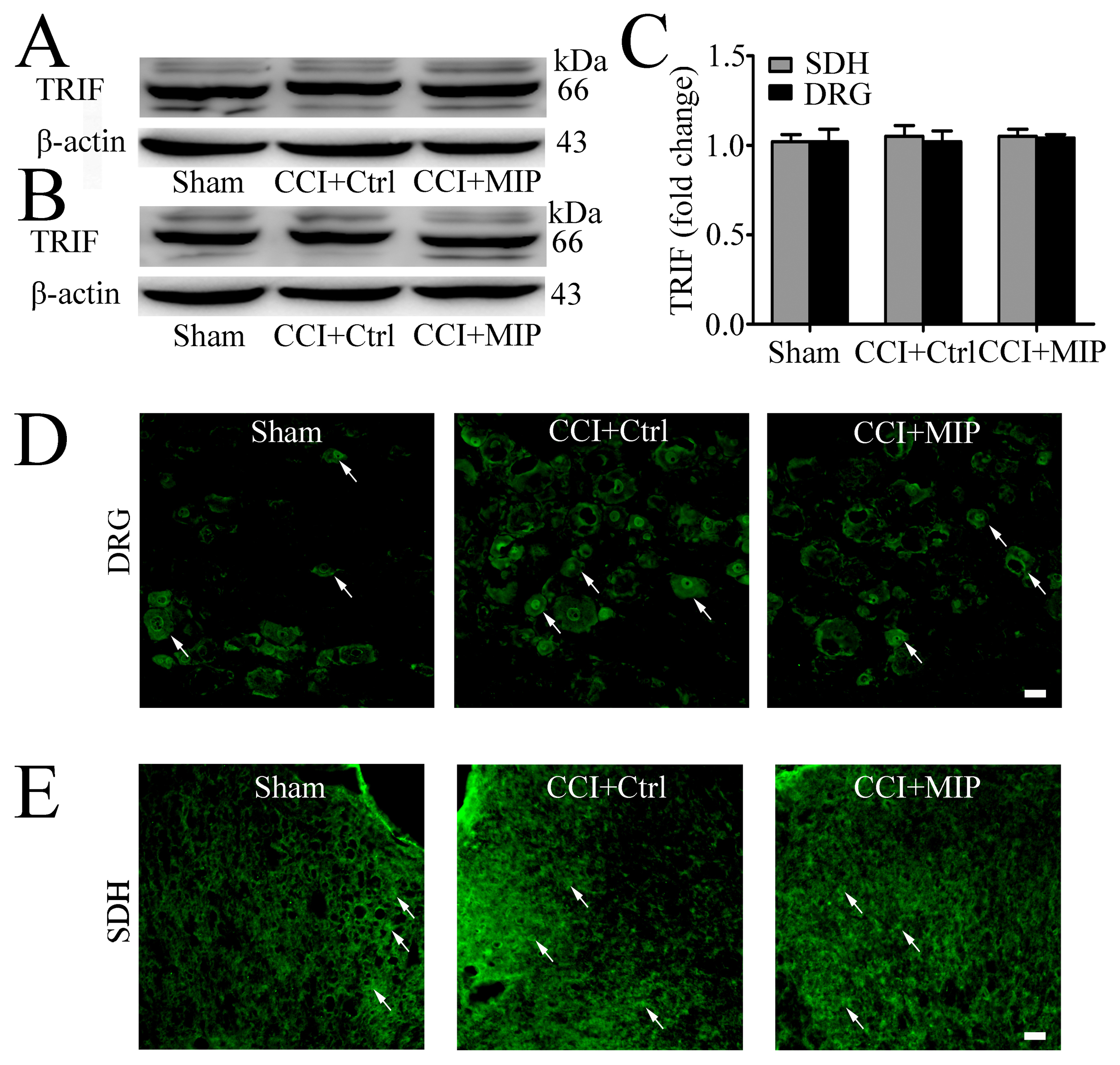
**

**Supplementary Figure S3** Protein level of TRIF by intrathecal administration of MIP in rat DRG and SDH after CCI. **(A-B)** Western blot shows MIP do not affect protein level of TRIF in DRG (A) and SDH (B). **(C)** Data summary of B. n = 4 in each group. No significant difference was found with One-way ANOVA test. **(D-E)** Immunofluorescence shows MIP have not inhibitory effects on expression of TRIF in DRG (D) and SDH (E). Scale bar: 20 μm. MIP (i.t., in a volume of 20μl, 500 mM) was given once a day on postoperative days 3, 4, and 5, respectively. The CP was used as control. Tissues were collected on postoperative days 14.


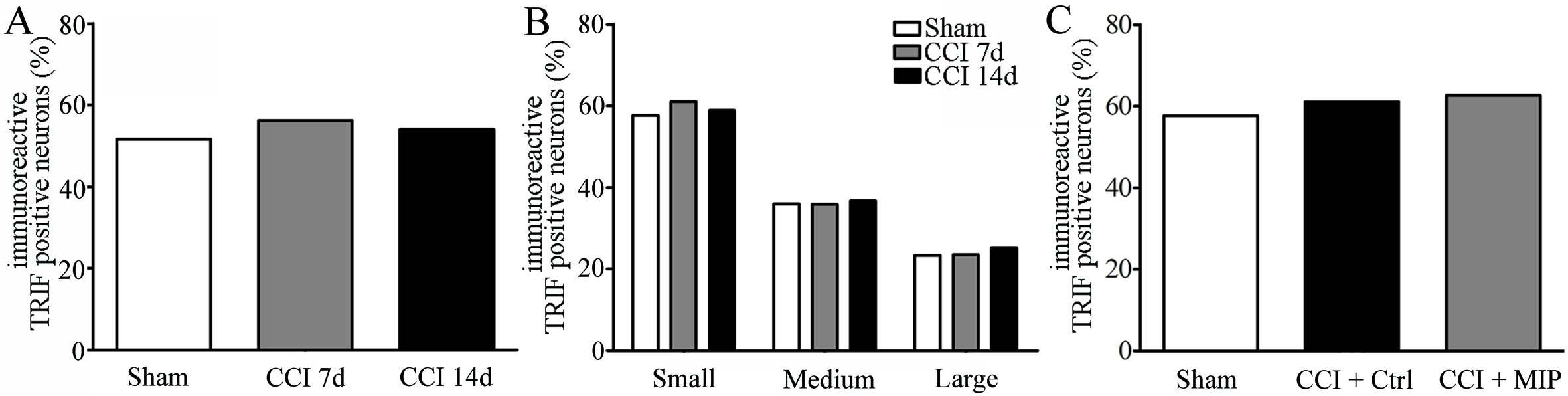


**Supplementary Figure S4** Quantification analysis from Immunofluorescence staining results of TRIF-positive neurons in DRGs. (A) Percentage of TRIF-positive neurons in DRGs of sham, CCI (7d) and CCI (14d) rats. (B) Size distribution of TRIF-positive neurons in DRGs of sham, CCI (7d) and CCI (14d) rats. (C) Percentage of TRIF-positive neurons in small-sized DRG neurons of sham, CCI + Ctrl and CCI + MIP rats. N = 4 in each group. No significant difference was found with Chi-square test in A-C (n > 100 neurons from 4 rats in each group)**.**
